# Diabetic Macular Edema Treated with 577-nm Subthreshold Micropulse Laser: A Real-Life, Long-Term Study

**DOI:** 10.3390/jpm11050405

**Published:** 2021-05-13

**Authors:** Luisa Frizziero, Andrea Calciati, Tommaso Torresin, Giulia Midena, Raffaele Parrozzani, Elisabetta Pilotto, Edoardo Midena

**Affiliations:** 1 Department of Neuroscience—Ophthalmology, University of Padova, 35128 Padova, Italy; lfrizziero@gmail.com (L.F.); andrear.cal@gmail.com (A.C.); tommasotorresin@gmail.com (T.T.); raffaele.parrozani@unipd.it (R.P.); elisabetta.pilotto@unipd.it (E.P.); 2 IRCCS—Fondazione Bietti, 00120 Rome, Italy; giulia.midena@gmail.com

**Keywords:** subthreshold micropulse laser, 577-nm laser, laser fixed parameters, diabetic retinopathy, diabetic macular edema, optical coherence tomography, autofluorescence, real-life

## Abstract

The aim of this study was to evaluate the long-term efficacy and safety of 577-nm subthreshold micropulse laser (SMPL) treatment in a large population of patients affected by mild diabetic macular edema (DME) in a real-life setting. We retrospectively evaluated 134 eyes affected by previously untreated center-involving mild DME, and treated with 577-nm SMPL, using fixed parameters. Retreatment was performed at 3 months, in case of persistent retinal thickening. Optical coherence tomography (OCT), along with short and near-infrared fundus autofluorescence, were used to confirm long-term safety. At the end of at least one year follow-up, a significant improvement in visual acuity was documented, compared to baseline (77.3 ± 4.5 and 79.4 ± 4.4 ETDRS score at baseline and at final follow-up, respectively), as well as a reduction in the mean retinal thickness of the thickest ETDRS macular sector at baseline. A reduction in the central retinal thickness and the mean thickness of the nine ETDRS sectors was also found, without reaching statistical significance. No patients required intravitreal injections. No adverse effects were detected. This study suggests that 577-nm SMPL is a safe and repeatable treatment for mild DME that may be applied to real-life clinical settings using fixed parameters and protocols.

## 1. Introduction

The World Health Organization has estimated that about 460 million people worldwide are affected by diabetes mellitus, and both the number of cases and the prevalence of diabetes have been steadily increasing over the past few decades [1]. Despite considerable progress made in recent decades in understanding its pathogenesis and implementing effective therapeutic strategies, diabetic retinal involvement, affecting approximately one-third of diabetic subjects, remains a major public health problem with important socioeconomic implications [1]. Diabetic macular edema (DME) is the earliest and most common cause of visual loss in patients with DR [1,2]. Although VEGF has been found to be a key molecule to the development of macular edema, DME is multifactorial and there are numerous potential biochemical pathways involved in its pathogenesis, such as inflammation [3]. Intravitreal anti-VEGF (Aflibercept, Ranibizumab and Bevacizumab) and steroids (Dexamethasone and Fluocinolone implant) injections are the current standard of care in DME treatment; however, they cause a significant burden for both the patients and the health care system because of the necessity of retreatments and follow-ups [4]. Moreover, some patients respond incompletely, or are complete non-responders to anti-VEGF injections. Lastly, the safety of intravitreal corticosteroids has limitations in many countries [5].

The Early Treatment Diabetic Retinopathy Study (ETDRS) demonstrated the effectiveness of macular laser photocoagulation in preventing progressive visual loss in eyes affected by DME [6]. However, side effects related to external retina necrosis in treated areas moved this treatment modality to limited cases, mainly with extra-foveal thickening [7]. Subthreshold micropulse laser (SMPL) combines the value of subthreshold treatment with micropulse technology to produce subthreshold retinal changes (invisible to any current imaging and functional diagnostic technology) without any evidence of retinal damage [8]. Yellow 577-nm SMPL has recently gained increasing diffusion in clinical practice. However, at present, standard and widely accepted indication criteria, setting parameters and treatment protocols to apply in clinical practice are still lacking [4,9,10].

Therefore, the aim of this study was to evaluate the long-term efficacy and safety of 577-nm SMPL treatment in a large population of patients affected by mild DME in a real-life clinical setting using a fixed setting of laser parameters, follow-up and retreatment modalities.

## 2. Materials and Methods

### 2.1. Study Population

This was a retrospective longitudinal study. Patients were consecutively enrolled among those who underwent SMPL for DME involving the foveal area between January 2017 and December 2019. A written consent form was obtained from all patients, as well as the approval from our institutional ethics committee (protocol number 3194/AO/14).

The research was carried out in accordance with the Declaration of Helsinki. The clinical records of all identified subjects were retrospectively reviewed. Inclusion criteria were: type 1 or 2 diabetes mellitus (DM) with good metabolic control (HbA1C < 8%), previously untreated center-involving mild macular edema with central retinal thickness (CRT) ≤ 400 μm and a follow-up period, after the first SMPL treatment, of at least 12 months [11]. Exclusion criteria were: proliferative DR, vision-limiting ocular conditions other than DR (such as amblyopia, age-related macular degeneration, myopic degeneration, retinal dystrophies, optic neuropathies, retinal vascular diseases, advanced glaucoma or corneal opacity of any cause), CRT > 400 μm and/or a history of previous macular laser treatment, previous intravitreal injection therapy, history of previous ocular trauma or surgery, except for uncomplicated cataract extraction.

Patients underwent a full ophthalmological evaluation at baseline and every three months after the first SMPL treatment, including spectral domain-optical coherence tomography OCT (SD-OCT) and fundus autofluorescence (FAF) using Spectralis (Spectralis HRA+OCT, Heidelberg Engineering, Heidelberg, Germany). Short-wavelength and near-infrared FAF were performed on a 30° field of view centered onto the fovea, as previously described [12]. Briefly, images were taken with a confocal scanning laser ophthalmoscope. The images’ resolution was 768 × 768 pixels. Argon laser light (488 nm) was used for short-wavelength FAF. A band-pass filter with a cut-off at 500 nm, included in the system, was inserted in front of the detector. A diode infrared laser light (790 nm) was used for near-infrared FAF. A band-pass filter with a cut-off at 830 nm, included in the system, was inserted in front of the detector [12]. An OCT macular map scan pattern was used, with a 20° × 20° scan area centered onto the fovea. Forty-nine horizontal scans 120 μm apart from each other were obtained in an automated pattern. At each follow-up examination, the follow-up modality was enabled, allowing for the examination to be repeated according to the previous baseline examination.

Retinal thickness was automatically calculated by the device in nine ETDRS sectors: a 1 mm diameter circular zone centered on the fovea (CRT) with two concentric outer rings of 3 and 6 mm diameters, each divided into 4 sectors. Mean total retinal thickness was recorded in each of the nine ETDRS sectors.

### 2.2. Laser Treatment Delivery

Prior to treatment, topical anesthesia with proparacaine 0.5% drops was administered. Yellow micropulse laser was delivered through an Ocular Mainster Focal/Grid lens (Ocular Instruments, Washington, DC, USA) using the Iridex IQ 577 (Iridex Corporation, Mountain view, CA, USA) instrument, with the following standard settings: spot size of 100 μm, power of 250 mW, duration of each spot 200 ms and 5% duty cycle, as previously described [9,13]. Confluent, non-overlapping spots, were applied over the whole macular area. Retreatment was performed every 3 months, according to the persistence of intraretinal fluid.

### 2.3. Statistical Analysis

The study parameters were analyzed using the usual descriptive statistic indicators: mean and standard deviation for quantitative variables, and absolute and relative (percentage) frequency for qualitative ones.

The following result parameters were considered in the statistical analysis of retinal thickness values gathered from the OCT exam: thickness of the central sector, mean thickness (arithmetic mean of 9 ETDRS sectors) and thickness of the sector that had the highest value at baseline.

Variations from the baseline to the end of follow-up were evaluated for each parameter by means of an ANOVA model adjusted for total follow-up length and the replication of measurements for patients who contributed with both eyes.

Then, the subgroups of eyes observed at different follow-up intervals were used to analyze the relationship between retinal thickness (the three parameters separately) and the number of laser treatments in relation to the length of follow-up. This analysis was performed by means of a multivariate linear regression model using retinal thickness as a dependent variable (one model for each parameter previously mentioned), and the number of laser treatments and the interval time as independent variables. Such models were adjusted for replication of measurements in patients who contributed with both eyes. The relationship was judged as noteworthy when the regression coefficient of the number of treatments variable resulted in statistical significance. Because of replication of testing, Bonferroni correction criterion was applied.

All the analyses were performed using SAS© 9.4 software (SAS Institute, Cary, NC, USA).

## 3. Results

A total of 134 eyes of 94 patients were enrolled. Mean diabetes duration was 21.2 ± 13.7 years and mean HbA1c was 7.5 ± 1.1 at baseline. Diabetic retinopathy was mild to moderate in 120 eyes and severe (for the presence of intraretinal microvascular abnormalities) in 14 eyes. Mean age at the beginning of follow-up was 66.7 ± 9.6 years. Patients’ clinical and demographic characteristics are summarized in Table 1.

**Figure 1:**
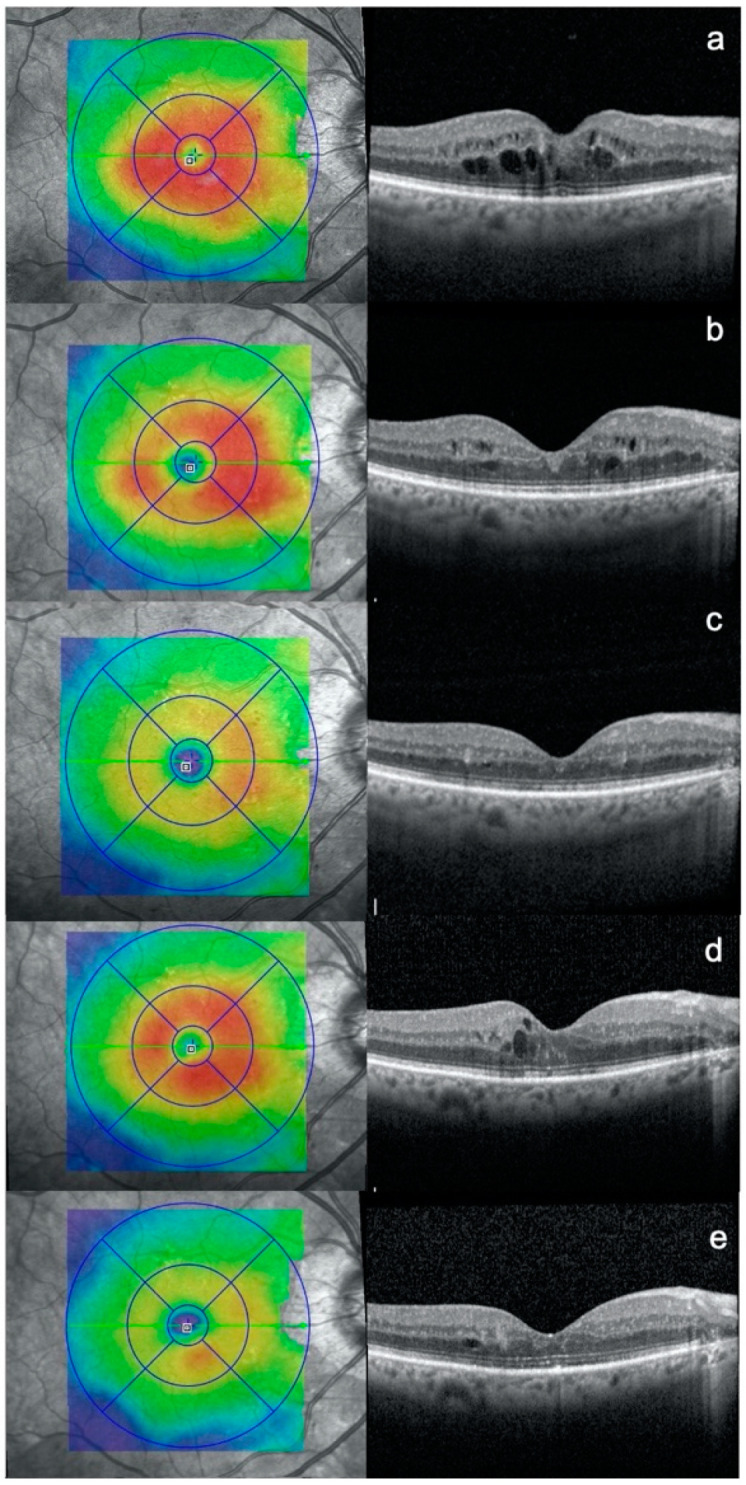
Optical coherence tomography (OCT) scans of a patient affected by diabetic macular edema: at (**a**) baseline, (**b**) 3 months after subthreshold micropulse macular laser (SMPL) 1st treatment, (**c**) 3 months after SMPL 2nd treatment, (**d**) additional 3 months after SMPL 2nd treatment, (**e**) 3 months after SMPL 3rd treatment.

Mean follow-up duration was 16.6 ± 6.5 months with an average of 2.3 ± 1.3 laser treatments per patient. At the end of follow-up, no significant change in HbA1c was detected. BCVA was 79.4 ± 4.4 (*p* < 0.001 compared to baseline) and a reduction of CRT and mean thickness of the nine ETDRS sectors was found, compared to baseline, without reaching statistical significance (−4.4 ± 49.5 μm, *p* = 0.3009 and −2.4 ± 22.9 μm, *p* = 0.235 for CRT and the nine sectors’ mean retinal thickness, respectively). A significant reduction of the mean retinal thickness of the thickest ETDRS sector at baseline was documented (−12.7 ± 36.2 μm, *p* < 0.0001) (Table 2, Figure 1). This reduction was significant at any follow-up visit.

A significant relationship was also found between a higher number of treatments and final lower thickness value at longer follow-up, for both mean retinal thickness in the nine ETDRS sectors and retinal thickness of the thickest sector at baseline (for longer follow-ups). Moreover, a greater number of laser treatments was significantly correlated with greater reduction in thickness in the thickest sector at baseline. After Bonferroni correction, significance was maintained for the relationship with the retinal thickness of the thickest sector at baseline. The analysis of laser effect on retinal thickness is summarized in Table 3 and Table 4.

OCT imaging, analyzed layer by layer, showed no laser side effects and no signs of outer retinal structure damage. Short-wave and near-infrared FAF acquired on a 30° field centered onto the fovea did not show any laser-related lesions in our study population, nor did the ophthalmoscopic fundus examination.

## 4. Discussion

The current devices for SMPL treatment are the infrared (810-nm) diode laser and the yellow laser, which emits at 577-nm [9,14]. Both wavelengths have been safely employed in treating patients affected by center-involving DME and comparable efficacy and safety have been reported [9,14]. The cornerstones of subthreshold retinal laser therapy were established and defined using an 810-nm near-infrared micropulse laser [15,16]. The 577-nm yellow laser was later introduced and, although at present it is the most commercially available device, its efficacy has been evaluated by fewer studies involving smaller sample sizes and shorter follow-up periods [9,13,15,16,17,18,19,20,21,22]. At present, a wide array of different duty cycle durations, spot sizes and power settings are employed by different clinicians, limiting, in our opinion, the diffuse clinical application of this technique [4,9,10,19]. This is a long-term follow-up retrospective report of patients treated with 577-nm SMPL for mild DME, with the aim of assessing the long-term morphologic efficacy and safety of repeated SMPL treatments using a fixed setting of laser parameters and follow-up and retreatment modalities.

A recent analysis compared fixed parameters treatment and titrated treatment for 577-nm yellow laser: the first one was recommended based on its greater speed of setup and the implicit avoidance of any possibility of titration errors [13]. Titration has also been discouraged due to the absence of any reliable, safe and scientifically-validated titration algorithms [23]. Fixed, previously validated parameters have been employed for all eyes included in this study, namely: 5% duty cycle, 200 μs duration, 250 mW power and 100 μm spot size [9,13]. Moreover, the whole macular area was treated in order to obtain a widespread “mass effect” involving the different cellular elements strictly correlated to each other in the macula, as suggested by Lavinsky et al. [14,24,25]. In fact, a correctly performed subthreshold micropulse laser treatment produces targeted metabolic retinal effects, without any necrotic (useless) effect [16]. Therefore, an extensive and confluent treatment is required to obtain a “mass effect” of cellular deactivation/activation [14,24]. No side effects have been reported both at OCT and FAF for all treated eyes.

As regards treatment indications, the efficacy of SMPL in severe edema has proven to be limited, possibly owing to the scattered distribution of laser energy throughout the target tissues [18,26]. Therefore, all treated eyes in this study were affected by mild DME with CRT inferior or equal to 400 μm [26]. Moreover, previous studies showed that the first morphological and functional results after SMPL appear at about three months [20]. Luttrull et al. found that macular thickness did not change significantly in the first 2 months after treatment. At 3 months, however, reduction of macular thickness was observed [27]. Lavinsky et al. reported a significant increase in BCVA from the third month after high-density SMPL [25]. A significant increase in central retinal sensitivity was also reported from the third month of follow-up after SMPL treatment [9]. Furthermore, in these studies, patients continued to improve over a long-term follow-up period (1 year) [21,25]. Therefore, at present, SMPL might be recommended in mild DME, with retreatment at least 3 months from the previous SMPL session.

Analyzing the functional and morphological results of our large number of patients treated with these standardized protocols and parameters, we found, at the end of the follow-up period (13.6 ± 6.5 months), improvement in visual acuity compared to baseline, which is associated with a reduction of retinal thickness that became significant only when considering the thickest ETDRS sector at baseline. This may be explained by the higher sensitivity of this last analysis: in case of mild edema, significant thickness variations may be difficult to detect, since a DME-related increase in thickness does not always affect the whole macular area and subsequent modifications can be “drowned out” by the relatively stable (by virtue of their already small baseline thickness) surrounding sectors, which will not shrink beyond a certain point. Considering the retinal thickness of the thickest ETDRS sector at baseline may allow for easier detection of thickness changes in the area that is most involved by the pathologic process at the beginning of the observation period. Moreover, these results suggest that the treatment of the whole macular area allows for the normalization of the most affected sectors and the stabilization of the other sectors, which are maintained over a long-term follow-up period.

Continuous improvement over a long-term follow-up period has already been suggested after SMPL treatment; moreover, we found that the number of treatments is also correlated to a greater reduction in macular thickness, and the relationship between retinal thickness and number of laser treatments increased in magnitude and statistical significance with the length of the observation period [21,25]. This result may be attributed to the slow and complex effect of SMPL that involves a multitude of retinal elements: repeating the treatment probably reinforces the normalizing process in the targeted retinal tissue and may be done without any significant safety concerns [21,28]. In the early stages of its application, it was speculated that the main target for SMPL action was RPE, and that the principal advantage of micropulse over continuous-wave lasers was the sparing of retinal and choroidal tissue adjacent to RPE [8,29]. More recently, aqueous humor (AH) analysis in eyes treated with 577-nm yellow SMPL for DME showed a significant reduction in AH concentration of chemotactic and pro-inflammatory cytokines after SMPL, as well as of specific Müller-cells related proteins (namely GFAP and Kir 4.1) [21,28]. Müller cells play a crucial role in the maintenance of the physiological retinal structure and function; they undergo activation and proliferation in response to different kinds of stressors and retinal injuries, including oxidative damage and hyperglycemia, and release pro-inflammatory and vaso-active substances that contribute to local inflammation and vascular permeability increase [30,31]. A reduction in retinal thickness accompanied by fluid reabsorption can also be related to a de-activating effect on Müller cells [20]. Thus, SMPL probably initiates a wide curtailing of local inflammatory processes that is accompanied by cellular de-activation and return to morphological integrity, with the restoration of the retinal microvascular network, as evidenced by a reduction of the foveal avascular zone area in the deep capillary plexus [22]. In this study, we did not analyze macular and peripheral perfusion status. Previous studies have reported the efficacy of both intravitreal steroids and anti-VEGF in reducing DME (and peripheral non-perfusion), regardless of the peripheral retinal perfusion [32,33]. However, further studies are needed to investigate the influence of retinal perfusion in mild DME and SMPL treatment. In conclusion, SMPL seems to cause a slow and generalized restoration of retinal homeostasis that includes blood-retinal barrier repair, reduction of local inflammation and widespread cellular normalization over the long term, as confirmed by our morphological results. All patients had good glycemic control and HbA1c remained unchanged during the study, showing that changes in mild DME were unrelated to the metabolic control.

Another noteworthy result is that, over the course of the follow-up period, no patients required intravitreal therapy. This confirms the previously reported potential for SMPL to control intraretinal fluid and alleviate the burden of intravitreal treatments [4,34,35]; SMPL was effective in keeping retinal thickness under control, stabilizing macular retinal homeostasis. A recent study has shown that visual acuity (in term of loss of five or more ETDRS letters) is not significantly different among eyes initially managed with intravitreal aflibercept, laser photocoagulation or observation at 2 years’ follow-up, in patients with good visual acuity (>79 ETDRS score) at baseline [36]. However, the proportion of eyes with visual acuity of 20/20 or better was significantly greater with aflibercept than observation, but not with laser photocoagulation, and eyes in the laser photocoagulation group had a lower likelihood of receiving aflibercept injections compared to eyes in the observation group [36]. This difference seems to suggest a possible benefit of laser photocoagulation in reducing the need for anti-VEGF. As already mentioned, SMPL proved to be as effective as laser photocoagulation, without its adverse effects (macular scarring and visual scotomas) [37]. Therefore, SMPL may have a role as an effective long-term retinal maintenance therapy, including in patients with good visual acuity, minimizing the risks and side effects associated with laser photocoagulation and intravitreal injections.

With regards to safety, OCT analysis did not detect any sign of disruption of the outer retinal layers’ integrity following 577-nm SMPL treatment; short-wave and near-infrared FAF images failed to disclose any secondary effects of the laser treatment on the macular area, and no visible result of the applied laser spots was apparent on ophthalmoscopy. No harmful effect of the laser treatment was detected in any eye over the course of follow-up, irrespective of the number of repeated laser treatments or total observation time.

The main limitation of this study is its retrospective nature. However, this was a real-life report on a large population of patients with mild center-involving DME, all treated by 577-nm SMPL with fixed parameters and retreatment modalities. It allowed us to prove the efficacy of treatment repetition over a long-term follow-up period, using a standardized treatment on the whole macular area. This may suggest the necessity of planning long-term treatment protocols with repeated SMPL sessions at pre-planned intervals of at least 3 months. This approach would also limit the need for control examinations, reducing the burden for patients and the health care system, and thus improving adherence to the treatment schedule.

## Figures and Tables

**Table 1:** Patients’ clinical and demographic characteristics at baseline.

N. eyes/patients	134/94
Age, years, mean ± SD	66.7 ± 9.6
Sex, F:M	33:61
Type of diabetes, 1:2	21:73
BCVA, ETDRS score ± SD	77.3 ± 4.5
Diabetes duration, years ± SD	21.2 ± 13.7
HbA1c, mean ± SD	7.5 ± 1.1
Follow-up, months, mean ± SD	16.6 ± 6.5

N: number; F: female; M: male; SD: standard deviation; BCVA: best corrected visual acuity; ETDRS: early treatment diabetic retinopathy study.

**Table 2:** Morphologic results (whole follow-up).

	Baseline Thickness (μm, Mean ± SD)	End of Follow-Up Thickness (μm, Mean ± SD)	Thickness Variation (μm, Mean ± SD)	*p*-Value
CRT	309.2 ± 45.8	304.7 ± 54.6	−4.4 ± 49.5	0.3009
RetMean	327.2 ± 21.3	324.9 ± 27.2	−2.4 ± 22.9	0.2358
RetMax	373.3 ± 34.2	360 ± 41.8	−12.7 ± 36.2	<0.0001

SD: standard deviation; CRT: Central retinal thickness; RetMean: mean retinal thickness in 9 ETDRS sectors; RetMax: retinal thickness in the thickest ETDRS sector at baseline.

**Table 3:** Morphologic results at follow-up intervals: mean retinal thickness in 9 ETDRS sectors.

Follow-Up Visit	Laser Treatments (Mean ± SD)	Retinal thickness Variation from Baseline (Micrometers, Mean ± SD (*p*-Value))	Effect of Number of Laser Treatments on Retinal thickness ^1^	Effect of Number of Laser Treatments on Retinal Thickness Variation ^2^
1	1	−2.7 ± 8.3 (0.0071)	−0.6925 (0.7204)	1.5149 (0.2945)
2	1.4 ± 0.7	−3.0 ± 13.9 (0.0246)	−3.5751 (0.0663)	−1.3270 (0.5054)
3	1.7 ± 0.8	−2.1 ± 19.7 (0.2283)	−2.0692 (0.1096)	−2.1302 (0.3151)
4	1.9 ± 0.9	−1.3 ± 23.1 (0.5009)	−1.2346 (0.2912)	−2.7467 (0.2051)
5	2.0 ± 1.0	−1.3 ± 23.2 (0.5273)	−1.5412 (0.1260)	−2.9853 (0.1286)
6	2.2 ± 1.2	−2.0 ± 22.7 (0.3015)	−1.5063 (0.0645)	−2.5123 (0.1316)
7	2.2 ± 1.2	−2.2 ± 22.7 (0.2706)	−1.4373 (0.0635)	−2.2309 (0.1653)
8	2.2 ± 1.3	−2.3 ± 22.8 (0.2504)	−1.5357 (0.0370)	−2.2271 (0.1500)
9	2.3 ± 1.3	−2.4 ± 22.9 (0.2358)	−1.5711 (0.0309)	−2.2756 (0.1397)

^1^ Regression coefficients (*p*-value) of multivariate linear regression model adjusted for repeated measures on the same eye. ^2^ Regression coefficients (*p*-value) of multivariate linear regression model adjusted for baseline value of retinal thickness. Statistically significant *p*-values according to Bonferroni adjustment due to multiplicity testing are reported in bold characters.

**Table 4:** Morphologic results at follow-up intervals: thickest sector at baseline.

Follow-Up Visit	Laser Treatments (Mean ± SD)	Retinal Thickness Variation from Baseline (Micrometers, Mean ± SD (*p*-Value))	Effect of Number of Laser Treatments on Retinal Thickness ^1^	Effect of Number of Laser Treatments on Retinal Thickness Variation ^2^
1	1	−9.8 ± 22.4 **(0.0005)**	3.7394 (0.4313)	9.7915 (0.0099)
2	1.4 ± 0.7	−10.2 ± 28.9 **(0.0003)**	−1.2129 (0.7553)	2.5570 (0.5176)
3	1.7 ± 0.8	−10.3 ± 34.5 **(0.0008)**	−3.8937 (0.0929)	−1.4021 (0.6968)
4	1.9 ± 0.9	−10.4 ± 35.9 **(0.0010)**	−3.3133 (0.0816)	−3.3168 (0.3137)
5	2.0 ± 1.0	−9.8 ± 36.1 **(0.0020)**	−3.3935 (0.0430)	−4.1176 (0.1761)
6	2.2 ± 1.2	−12.1 ± 35.8 **(0.0001)**	−4.6878 **(0.0005)**	−5.6862 (0.0288)
7	2.2 ± 1.2	−12.7 ± 36.2 **(<0.0001)**	−4.9631 **(<0.0001)**	−5.7742 (0.0219)
8	2.2 ± 1.3	−13.1 ± 36.5 **(<0.0001)**	−5.2394 **(<0.0001)**	−5.8335 (0.0163)
9	2.3 ± 1.3	−13.3 ± 36.5 **(<0.0001)**	−5.1858 **(<0.0001)**	−5.6725 (0.0188)

^1^ Regression coefficients (*p*-value) of multivariate linear regression model adjusted for repeated measures on the same eye. ^2^ Regression coefficients (*p*-value) of multivariate linear regression model adjusted for baseline value of retinal thickness. Statistically significant *p*-values according to Bonferroni adjustment due to multiplicity testing are reported in bold characters.

## Data Availability

The data presented in this study are available in the article. Eventual additional data are available on request from the corresponding author.
